# A philosophical defence of limited foreknowledge open theism

**DOI:** 10.1007/s11153-025-09961-5

**Published:** 2025-07-22

**Authors:** Marcus Ackermann

**Affiliations:** Faculty of Philosophy, University of Cambridge, Sidgwick Avenue, Cambridge, CB3 9DA UK

**Keywords:** Open theism, Limited foreknowledge, Logical fatalism, Grounding, Branching time, Thin red line

## Abstract

Limited foreknowledge open theism (LFOT) is the view that there are contingent truths about the future but that even an omniscient God cannot foreknow them. This paper mounts a three-pronged philosophical defence of this doctrine. On the one hand, I will show that it can be given a formal model and semantics that is both *formally* and *descriptively* adequate. In doing so, I draw heavily on the most recent advancements in Thin Red Line semantics, and ultimately recommend a framework that combines familiar formal elements in a novel way: this places LFOT on a solid logical foundation. On the other hand, I will show that the two most prominent objections to LFOT, namely Todd’s (Philosophia 42(2):523–538, 2014) *grounding objection* and Arbour's (Int J Philos Relig 73:189–207, 2013) *fatalistic objection*, can both be met. With respect to the former, we will see that the proponent of LFOT is (*pace* Todd) able to heed a suitable grounding requirement for future contingent truths. And as regards the latter, it will be demonstrated (*pace* Arbour) that LFOT does not entail logical fatalism. The upshot of this is that LFOT is, at least from a philosophical perspective, perfectly plausible.

## Introduction

Ever since Prior (1967) created the formal framework of *branching time*, there has been a venerable tradition of using it to investigate the familiar tension between divine foreknowledge and creaturely freedom. It has been utilised to formalise famous *compatibilist* views of (amongst others) Anselm, Ockham and Leibniz (Øhrstrøm, 1984) or de Molina (Restall, 2011; De Florio and Frigerio, 2020). However, little attention has been paid to formally examining *incompatibilist* views. I hope to redress this neglect. More specifically, this paper will scrutinise *limited foreknowledge open theism* (LFOT), an incompatibilist view which affirms that there are truths about the contingent future but denies that these are known to even an omniscient God (Todd and Fischer, 2015). Three arguments will be made.

Firstly, I will argue that temporal reality as suggested by LFOT can be formally represented by a novel version of the theory of the Thin Red Line (TRL). This theory holds that even assuming indeterminism there is such a thing as an *actual future* (Malpass and Wawer, 2012). Formally, it is usually developed in a branching time framework featuring a representation of the actual future – a metaphorical thin red line (trl). (A brief note on terminology: I use the lower-case “trl” to refer to the notion of an actual future itself, and the uppercase “TRL” to refer to theories that employ this notion in a branching framework.) Secondly, I will show that making this formal connection affords the resources to respond to Todd’s (2014) *grounding objection* to LFOT in two different ways. Thirdly, I will offer a rebuttal to Arbour’s (2013) *fatalistic objection* to LFOT. Given that these are the two most prominent^1^ philosophical objections to LFOT, and seeing how a formalisation of LFOT is desirable both for its own sake and instrumentally useful, this paper is therefore aptly called a defence (though not an endorsement) of LFOT.

I first outline an argument for the incompatibility of foreknowledge and freedom (Sect. 2) to set the stage for introducing LFOT (Sect. 3). I then introduce the framework of branching time (Sect. 4) before considering and rejecting two *prima facie* attractive TRL views from the literature, namely the *history-independent* (Sect. 5) and the *postsemantic* TRL (Sec. 6). Next, I propose and defend a novel hybrid view that I call the *history-dependent* TRL (Sect. 7). Finally, I deal with Todd’s (2014) objection (Sect. 8) and Arbour’s (2013) objection (Sect. 9) before concluding (Sect. 10).

## The divine foreknowledge argument

There are many ways to specify the argument for an incompatibility between divine foreknowledge and creaturely freedom. The one I will present is based on Pike’s (1965) seminal treatment of this topic (cf. also Fischer and Tognazzini, 2014). Suppose that God 1000 years ago held the true belief that you would read this paper today.^2^ Given the infallibility of divine knowledge, it follows that in order to do otherwise than read this paper today, you would have to be able to make it the case that God did not truly believe this. That is, you would have to be able to act in a way such that, were you to act that way, either (i) God would have held a false belief, (ii) God would have held a different belief in the past, or (iii) God would have not existed in the past. But you do not have any of these abilities. On the one hand, it is simply *logically impossible* for God to hold a false belief. And on the other hand, it is *metaphysically impossible* to act in such a way as described in (ii) and (iii) – the past is fixed, after all. Consequently, you cannot do otherwise than read this paper today. And the argument generalises. Nobody was, is, or will ever be able to act otherwise than they in fact do. But free will necessarily requires the ability to do otherwise. Consequently, free will was, is, and always will be an illusion. Call this the *divine foreknowledge argument* (DFA).

For now, two parts of the DFA require highlighting, both of which will become important again later. On the one hand, there is the *foreknowledge assumption*:

**Foreknowledge assumption.** For every true proposition *P* saying of a free creature that it will perform some action in the future, God has always believed that *P*.

On the other hand, there is the principle of the *necessity of the past*. Formulating it in an informative yet theoretically unbiased way is a hard task. At least two important choices have to be made. Firstly, what *type* of entity now-necessity (i.e. the kind of *historic* necessity at stake) is predicated of. The usual candidates here include propositions, facts, and events. And secondly, whether *all* past tokens of this type are now-necessary, or just some proper subset of them. A commonly drawn distinction here is that between *hard* and *soft* facts about the past (see Todd, 2013). For the present purpose, I will follow Hunt and Zagzebski (2022, §1) in predicating now-necessity of *all* past tokens of the type *event* (E), resulting in the following principle:

**Necessity of the past.** If E occurred in the past, it is now-necessary that E occurred then.

## Limited foreknowledge open theism

*Open theism* is an umbrella term for views which deny that God has foreknowledge of the contingent future (Todd and Fischer, 2015).^3^ It comes in two main varieties: *open future open theism* is the position that because there are no true future contingents there is nothing about the contingent future for even an omniscient being to know, whereas LFOT is the view that there are true future contingents which nonetheless are unknown to God (ibid.). LFOT has prominent defenders, including van Inwagen (2008), Swinburne (2006), and Hasker (1989). The latter’s exposition is the most detailed, so it is him I will focus on.

The argument for LFOT is that a denial of the DFA’s conclusion is more plausible than the conjunction of its premises, and that the foreknowledge assumption is the weak link. Hasker (ibid., p.69) observes that the assumption seems to follow from the idea that there exist truths about the contingent future in conjunction with the thesis that what it is to be omniscient is to, necessarily, believe all truths and not believe any falsehoods. He (ibid., p.73f.) then argues that this is too strong a definition of omniscience, suggesting that an omniscient being need not know all truths, but rather only those truths which it is *logically possible* for it to know. Finally, he (ibid.) then helps himself to the denial of the DFA’s conclusion. This means that one of the assumptions that led to the DFA’s conclusion has to go, and Hasker chooses the foreknowledge assumption. Here, I second Todd’s (2014, p.531f.) analysis that Hasker is best interpreted as suggesting that the foreknowledge assumption follows only from an *inflated* understanding of divine omniscience as requiring knowledge of every truth. Once this criterion is suitably relaxed, however, it is no longer obvious that God would foreknow the contingent future; after all, future contingent truths may be (or are, depending on how strong a claim one makes) amongst the truths that it is logically impossible to foreknow (ibid.).

This discussion gives two-fold content to a *first desideratum* for a satisfying formal account of LFOT, namely that of *descriptive adequacy*. On the one hand, this condition demands that a suitable formal framework be able to represent future contingency of the kind required for free action – after all, the proponent of LFOT affirms agential freedom. On the other hand, it calls for a sufficient notion of future contingent truth. Because the foreknowledge assumption is couched in the language of *propositions*, the requisite notion here is plausibly that there be a *comprehensive set of contingent propositional truths about the actual future* (where the qualifier “comprehensive” just indicates that, for *every* future-tensed proposition, either it or its negation is true). Based on these considerations, a framework that naturally commends itself to the proponent of LFOT is thus a *branching time model* (supposedly securing contingency) enriched by a *trl* (supposedly guaranteeing a relevant notion of truth).

A second desideratum, *formal adequacy*, makes less specific demands. As with any formal framework, meeting this desideratum (at least) requires giving a satisfactory account of the relevant operators and generating an acceptable set of validities. I will be measuring the success of candidate frameworks by reference to these two desiderata.

## Branching time

The idea behind *branching time structures* (BT structures) is to represent tempo-modal reality in a tree-like fashion, with a single “trunk” towards the fixed past and multiple “branches” towards the indeterministic future. Before being publicly developed in Prior (1967), this approach had initially been conceived of by Kripke and suggested to Prior in private correspondence (Ploug and Øhrstrøm, 2012).

Formally, a BT structure is an ordered pair $\Im =\langle M,\prec \rangle$, where *M* is a non-empty set of *moments* (*representations* of spatially maximal and temporally instantaneous possible slices of reality), and $\prec$ is a *strict partial order* on *M* (the earlier-than relation). This ordering is subject to the constraints of (BL) *backwards linearity* (meaning that there is no branching towards the past), (HC) *historical connectedness* (meaning that any two moments share a common lower bound in *M*), and (HI) *historical infinity* (meaning that there is no “last” moment). I use $m\preceq m'$ as shorthand for ($m\prec m'$ or $m=m'$).

### Definition 1

(BT structure) A BT structure $\Im =\langle M,\prec \rangle$ consists of a non-empty set *M* ordered by an irreflexive, asymmetric, and transitive relation $\prec$ such that

(BL) $$\forall m_1, m_2, m_3 \in M[(m_2\prec m_1\wedge m_3\prec m_1)\rightarrow (m_2\prec m_3\vee m_3\prec m_2$$ $$\vee m_2=m_3)]$$;
(HC) $$\forall m_1,m_2\in M \exists m_3\in M(m_3\preceq m_1\wedge m_3\preceq m_2)$$;
(HI) $$\forall m\in M\exists m'\in M(m\prec m')$$.

Given a BT structure $\Im$, a maximal chain of moments in *M* linearly ordered by $\prec$ is called a *history* *h*. Intuitively, histories represent possible courses of events, i.e. temporally complete ways reality could be. The set of all such histories in BT structure $\Im$ is written as $hist$($\Im$).

The language under consideration will be the tempo-modal propositional language $\mathcal {L}$. Its vocabulary consists of a set $Sent$ of atomic sentence letters (*p*, *q*, ...) whose content is wholly about the present, the primitive Boolean connectives $\lnot$ and $\wedge$, the temporal operators *F* (“It will be the case that...”) and *P* (“It was the case that...”), and the modal operators $\Box$ (“It must be the case that...”) and $\Diamond$ (“It might be the case that...”). The syntax of $\mathcal {L}$ is given by the following Backus-Naur-form:

$$\phi {:}{:}{=}p\in Sent\mid \lnot \phi \mid \phi \wedge \phi \mid F\phi \mid P\phi \mid \Box \phi \mid \Diamond \phi$$.

We can now introduce the notion of a *BT model*. This is an ordered pair $\mathcal {M}=\langle \Im ,V\rangle$ consisting of a BT structure $\Im$ and a *valuation function* $V: Sent \rightarrow \mathcal {P}(M)$ that assigns to each sentence letter of $\mathcal {L}$ a subset of *M* (intuitively, the set of moments at which the sentence letter is true).

### Definition 2

(BT model) A BT model is an ordered pair $\mathcal {M}=\langle \Im ,V\rangle$ such that $\Im$ is a BT structure and *V* a valuation function $V: Sent \rightarrow \mathcal {P}(M)$.

A recursive *semantics* for formal models tells us how to assign truth values to *sentences* at a *point of evaluation*. Here, a “sentence” is an abstract entity consisting of a string of words or symbols whose formation obeys the grammatical rules of a given language (Huang, 2007), and a “point of evaluation” is a non-empty set of parameters (MacFarlane, 2003). The most popular semantics for BT models, which I shall call *Priorian-Ockhamist* semantics, has the point of evaluation be a moment/history pair *m*/*h* (with $m\in h)$; this allows for a smooth interaction of modal and temporal operators and generates a pleasing set of validities (see Prior, 1967).

### Definition 3

(Priorian-Ockhamist semantics) Where $\mathcal {M}_{m,h}\vDash \phi$ is read as “$\phi$ is true at moment $m\in h$ relative to history *h* in BT model $\mathcal {M}$”,

$$\begin{array}{ll} -\mathcal {M}_{m,h}\vDash p & \text {iff } m\in V(p) \text { where }p\in Sent ;\\ - \mathcal {M}_{m,h}\vDash \lnot \phi & \text {iff }\,\mathcal {M}_{m,h}\nvDash \phi ;\\ - \mathcal {M}_{m,h}\vDash \phi \wedge \psi & \text {iff } \,\mathcal {M}_{m,h}\vDash \phi \text { and }\mathcal {M}_{m,h}\vDash \psi ;\\ - \mathcal {M}_{m,h}\vDash P\phi & \text {iff }\, \exists m'(m'\prec m\text { and }\mathcal {M}_{m',h}\vDash \phi );\\ - \mathcal {M}_{m,h}\vDash F\phi & \text {iff }\,\exists m'(m\prec m'\text { and }m'\in h\text { and }\mathcal {M}_{m',h}\vDash \phi );\\ - \mathcal {M}_{m,h}\vDash \lozenge \phi & \text {iff }\, \exists m'(m\prec m'\text { and }\mathcal {M}_{m',h}\vDash \phi );\\ - \mathcal {M}_{m,h}\vDash \Box \phi & \text {iff} \, \forall h'[(m\in h'\rightarrow \mathcal {M}_{m,h'}\vDash \phi )\end{array}$$.

So this semantics does not tell us what will happen as of a given moment *tout court*; instead, we always have to specify a history. But this is problematic because there is nothing in the model that privileges any of the histories. They are all as good as each other when it comes to evaluating the future tense operator at a moment. Consequently, this picture lacks a comprehensive *set of* contingent propositional truths about the *actual* future – all it can provide is truths about *this* or *that* future, none of which enjoy the privileged status of actuality. TRL frameworks promise to fix this issue.

In the following three sections I will argue that the only two variants of this approach that are *prima facie* promising for the present purpose, namely the history-independent TRL and the postsemantic TRL, are unavailable to the proponent of LFOT, the former because it fails the second desideratum (i.e. is formally inadequate), the latter because it fails the first desideratum (i.e. is descriptively inadequate). I will then present and defend a novel hybrid view that combines elements of both these approaches in a way that satisfies both desiderata.

## The history-independent TRL

The easiest way of introducing a trl into a BT model is by having the structure itself designate a single privileged history (see e.g. Øhrstrøm, 1981). The resulting $\hbox {TRL}_1$ model is thus an ordered triple $\mathfrak {M}=\langle \Im , V, {trl}_h\rangle$, where $\Im$ is a BT structure, *V* is a valuation function, and ${trl}_h$ is the unique actual history.

### Definition 4

($\hbox {TRL}_1$ model) A $\hbox {TRL}_1$ model is an ordered triple $\mathfrak {M}=\langle \Im , V, {trl}_h\rangle$ such that $\Im$ is a BT structure, *V* is a valuation function, and ${trl}_h\in hist(\Im )$.

A natural semantics for such models (see e.g. Wawer, 2014) eliminates the history-parameter from the point of evaluation and ties the interpretation of the future tense operator to ${trl}_h$: on this picture, a future-tensed sentence $F\phi$ is thus true at a moment *m* if and only if there is some moment $m'$ later than *m* such that $m'$ lies on ${trl}_h$ and $\phi$ is true at $m'$.

### Definition 5

($\hbox {TRL}_1$ semantics) Where $\mathfrak {M}_{m}\vDash \phi$ is read as “$\phi$ is true at moment *m* in $\hbox {TRL}_1$ model $\mathfrak {M}$”,

$$\begin{array}{ll} -\mathfrak {M}_{m}\vDash p& \text {iff } m\in V(p) \text { where }p\in Sent ;\\ - \mathfrak {M}_{m}\vDash \lnot \phi & \text {iff }\mathfrak {M}_{m}\nvDash \phi ;\\ - \mathfrak {M}_{m}\vDash \phi \wedge \psi & \text {iff }\mathfrak {M}_{m}\vDash \phi \text { and }\mathfrak {M}_{m}\vDash \psi ;\\ - \mathfrak {M}_{m}\vDash P\phi & \text {iff }\exists m'(m'\prec m\text { and }\mathfrak {M}_{m'}\vDash \phi );\\ - \mathfrak {M}_{m}\vDash F\phi & \text {iff }\exists m'(m\prec m'\text { and }m'\in {trl}_h\text { and }\mathfrak {M}_{m'}\vDash \phi );\\ - \mathfrak {M}_{m}\vDash \Diamond \phi & \text {iff }\exists m'(m\prec m'\text { and }\mathfrak {M}_{m'}\vDash \phi );\\ - \mathfrak {M}_{m}\vDash \Box \phi & \text {iff }\forall h[(m\in h\rightarrow \exists m'(m'\in h\text { and }m\prec m'\text { and }\mathfrak {M}_{m'}\vDash \phi )].\end{array}$$

But this semantics arguably fails the desideratum of formal adequacy. Because the interpretation of the future tense operator is tied to the actual history, all future tensed sentences (including tautologies) will come out false at non-actual moments, i.e. moments that do not lie on ${trl}_h$. But surely $F(\phi \vee \lnot \phi )$ should come out as true even at non-actual moments: this is because its truth has nothing at all to do with *actuality* and everything to do with *necessity* (cf. Malpass and Wawer, 2012, p.129). $\hbox {TRL}_1$ fails this requirement.

One way in which temporal logicians have responded to this formal shortcoming is by replacing the *constant* $trl_h$ in the model with a *trl function* $trl _{ fct }: M\rightarrow hist (\Im )$ that assigns a (quasi-)actual history to *every* moment (see e.g. McKim and Davis, 1976; Barcellan and Zanardo, 1999). I will not be considering this approach here, however, as it isn’t even *prima facie* a promising candidate for the present purpose. This is because it, on a very natural interpretation (see e.g. Restall, 2011; De Florio and Frigerio, 2020), commits one to there being true *counterfactuals of creaturely freedom* (i.e. true statements concerning what any free creature *would* do *were* it to find itself in any given circumstances). But the proponent of LFOT does not posit truths of this sort. She must thus look elsewhere.

## The postsemantic TRL

Some (e.g. Malpass and Wawer, 2012; Wawer, 2014; Iacona, 2014; Wawer and Malpass, 2020) have suggested that we can formally make good on the idea of there being an actual history *without* including it as an explicit element of our model: the general idea here is to return to a BT model as per Definition 2 equipped with standard Priorian-Ockhamist semantics as per Definition 3, and to introduce the notion of a privileged actual future via the notion of a *context (of utterance)*.

Let an “utterance” be a *concrete realisation* of a sentence, i.e. the use of a piece of language by a particular speaker (Huang, 2007). The context, then, is the concrete set of circumstances (usually taken to consist of at least place, time, speaker, and world) in which an utterance takes place. In orthodox *indexical* semantics à la Lewis (1980) and Kaplan (1989), the context is said to be necessary to supply the correct meaning for uttered indexical terms like “I” or “here” (whose correct interpretation will obviously depend on *who* the speaker is and *where* she is located). For technical reasons (see MacFarlane, 2003, p.328f.), however, one simply cannot give a *recursive* definition of *utterance-truth at a context*. One must therefore define utterance-truth *in terms of* a recursive notion of sentence-truth at a point of evaluation, and the role of the *postsemantics* is to tell us how to do that (ibid., p.329f.).

The precise idea behind the postsemantic TRL, then, is that the context provides both a moment-parameter $m_c$ (namely, the moment at which the utterance in question is made) as well as, and this is crucial, the relevant trl $h_c$ whence to begin our Priorian-Ockhamist semantic computation; borrowing generously from Wawer and Malpass (2020, p.2199), one can then give the following definition of TRL postsemantics:

### Definition 6

(TRL postsemantics) The sentence “$\phi$” is true at a context of utterance *c* iff $\phi$ is true at the point of evaluation $m_c$/$h_c$, where $m_c$ is the moment of the context and $h_c$ is the constant trl designated by the context. Where $\mathcal {M}$ is a BT model, $m_c\in M$, $h_c\in hist(\Im )$, and $c\Vdash \phi$ is shorthand for “the sentence $\phi$ is true at the context of utterance *c*”,

$$c\Vdash \phi$$ iff $$\mathcal {M}_{m{_{c}}/h{_{c}}}\vDash \phi$$.

In theory, a postsemantic TRL view need not always designate the same constant trl but could, akin to the trl-function view discussed at the end of Sect. 5, designate different trls at different moments. However, I will be assuming it is used in the former way, for the same sort of reason previously given against the trl-function view itself. Let us now consider an example. Suppose I now say “It will be the case that *p*”. On the view at stake, the context of my utterance then designates both a moment and the constant trl. With these in hand, we can now check the relevant semantic clause for *Fp* in the Priorian-Ockhamist semantics, using this moment and the relevant history as the parameters in our point of evaluation. Et voilà.

The problem with this approach, for the proponent of LFOT, is that it is *descriptively inadequate*. This is because the postsemantic TRL inherits the concern raised for Priorian-Ockhamist semantics in Sect. 4, namely that it too lacks a comprehensive set of contingent propositional truths about the actual future. On the one hand, propositions expressed by “unrealised” sentences are again true only relative to this or that history (and we have seen why this is unsatisfactory). On the other hand, propositions expressed by “realised” sentences may be actually true in the requisite sense, that is, there may be *some set* of contingent propositional truths about the actual future. But the problem here is that this set will fail to be *comprehensive*. After all, surely it isn’t the case that every true proposition about the contingent future is at some stage *uttered by a speaker*. On this picture, the proponent of LFOT thus winds up with *too few* relevant truths. Consequently, she must look elsewhere yet again.

## A hybrid view

As the previous sections show, securing the goal of descriptive adequacy requires there to be a trl that is part of the structure of our model rather than a stowaway smuggled in via the notion of a context. But in order to overcome the formal inadequacy of history-independent semantics for such models, we need to utilise a *history-dependent* semantics like Priorian-Ockhamism instead – only then is it guaranteed that we are able to give a satisfactory interpretation of the future tense operator even at non-actual moments. The two desiderata I put forward thus call for the proponent of LFOT to *combine* familiar elements (a branching model containing a trl as one of its elements, and Priorian-Ockhamist semantics) in a *novel* way (“novel” in the sense that, for reasons we will consider shortly, it has so far been shunned and to the best of my knowledge never seriously proposed). Let us call this view the history-dependent TRL. The remainder of this section will first introduce some more technical machinery required to fully elucidate this view before then offering a defence of this approach.

### Presemantics

The history-dependent TRL theorist *could* simply use a $\hbox {TRL}_1$ model $\mathfrak {M}=\langle \Im , V, {trl}_h\rangle$ as per Definition 4 (and combine it with Priorian-Ockhamist semantics instead of $\hbox {TRL}_1$ semantics). But for the present purpose, it will be beneficial to adopt a slightly more expansive model. This is because, as it stands, a given $\hbox {TRL}_1$ model does not contain the resources to express which *metaphysical presuppositions* about possibility and temporal ontology are baked into it. If one is a *branching concretist*, for example, one believes that the topology of our concrete world itself is that of an eternal and unchanging tree and that hence *every* moment in the model exists concretely; if, on the other hand, one is a *branching actualist*, one believes that our concrete world is either represented by or identical to a *single* history through the otherwise abstract tree of possibilities and that hence only moments on the trl represent concrete reality (or are themselves concrete) (Wawer, 2014). And even amongst branching actualists there may be disagreement about temporal ontology, i.e. disagreement about *how many* moments on the trl represent what concretely exists (or exist concretely themselves) (ibid.).

In order to sort out this mess, Wawer (ibid.) developed a tool he labelled the *presemantics*. Its purpose is to encode all the relevant metaphysical presuppositions via the familiar notion of a context. A presemantics contains two elements. Firstly, it contains a set *C* of *admissible* contexts *c* ordered via the *earlier-than or simultaneous-with* relation $\preceq$. An “admissible context” here is a technical term for a concrete environment that, if one *were* to add a speaker into it, *would* be the context of an utterance. Put simply, an admissible context differs from the regular notion of a context only in that it need not contain a speaker. Secondly, a presemantics contains a function *I* that tells us which admissible contexts are designated by which moments. The idea here is simple. We have seen that admissible contexts exist *concretely* by definition. Consequently, choosing which moments get matched with admissible contexts and which don’t is *ipso facto* to partition the set of moments into those that represent concrete reality (or exist concretely themselves) and those that do not. Being able to formally enshrine this information will come in handy when considering certain objections to LFOT, namely those objections where the range of replies is restricted by one’s relevant metaphysical presuppositions (as is the case with e.g. the grounding objection we will consider later).

The presemantics I am about to present differs from Wawer’s in one crucial way. He seemingly construes an admissible context as being either *represented* by, or *identical* to, certain moments; either ways, he posits a direct mapping of one onto the other.^4^ But this, I believe, gives us *too few* contexts. Remember that an admissible context need not involve a speaker, but at least involves a time, location, and world. So it seems natural to me to say that a temporally instantaneous slice of our concrete reality (or a representation thereof) designates as many admissible contexts as it contains locations (seeing how the time- and world-parameters are fixed).

More formally, let the *simultaneity relation* $\sim _{sim}$ (which falls out of the relation $\preceq$ for free) be an equivalence relation partitioning *C* into as many equivalence classes as there are moments on the trl. What Wawer calls an admissible context *c* is thus, on my view, best understood as one such equivalence class [*c*]. Let us therefore coin the technical term of a “temporal context” $[c]$ to refer to any such equivalence class. The set $C^t$ of temporal contexts is thus the quotient $C/\sim _{sim}$ of set *C* by equivalence relation $\sim _{sim}$. Crucially, this difference is more than mere notational variation – indeed, I believe my approach is essential to avoid under-counting contexts. So whereas for Wawer (ibid., p.379) the function *I* is “a map from the set of all contexts *C* to the set of moments of the model”, I define *I* as a function $I:C^t\rightarrow M$ from temporal contexts (equivalence classes of admissible contexts) to moments. Finally, we are now in a position to put this formalism to work.

I will set aside branching concretism for the present purpose. This is because it sits very uneasily with the idea that one amongst the many equally concretely existing futures should somehow be privileged (as the TRL suggests is the case). I will hence focus solely on branching actualism and distinguish three variants thereof. Further, for ease of exposition, I will make the assumption that moments on the trl *represent* temporal contexts rather than being identical to them – this is a natural choice for branching actualists (see ibid.). On the one hand, *presentist* branching actualism is the view that concrete reality is represented by a single moment on the trl. A presemantics for this view thus encodes the following information (cf. ibid., p.383):

#### Definition 7

(Presemantics for presentist branching actualism) There is only one equivalence class of admissible contexts ($\mid C^t\mid =1$) which *I* maps to the present moment $m\in M$.

*Eternalist* branching actualism, on the other hand, is the view that concrete reality is represented by the entire trl. It is encoded by the following presemantics (cf. ibid.):

#### Definition 8

(Presemantics for eternalist branching actualism) The set $C^t$ of temporal contexts is isomorphic to a single history $h\in hist (\Im )$ such that *I* maps a specific $[c]\in C^t$ to every moment $m\in h$.

Finally, *growing block* branching actualism is the view that concrete reality is represented by past and present moments on the trl. A suitable presemantics here looks as follows (cf. ibid.):

#### Definition 9

(Presemantics for growing block branching actualism) The set $C^t$ of temporal contexts is isomorphic to the set $GB _{m_p}=\{m\in M\mid m\preceq m_p\in M$} (where $m_p$ is the moment designated as present) such that *I* maps a specific $[c]\in C^t$ only to moments $m,m',...\preceq m_p$.

### The history-dependent TRL

With this in hand, we are now able to define a $\hbox {TRL}_2$ model by adding the new elements $C^t$ and *I* to the $\hbox {TRL}_1$ model familiar from Definition 4.

#### Definition 10

($\hbox {TRL}_2$ model) A $\hbox {TRL}_2$ model is an ordered quintuple $\mathfrak {M}^+=\langle \Im , V, trl_h, C^t, I\rangle$ such that $\Im$ is a BT structure, *V* is a valuation function, $trl_h\in hist (\Im )$, $C^t$ is the quotient $C/\sim _{sim}$ of set *C* by equivalence relation $\sim _{sim}$, and *I* is a function $I:C^t\rightarrow M$.

Where it is relevant which presemantics a model is supplied with, I will indicate this in the model's subscript: $\mathfrak {M}^+_e$ for eternalist branching actualist presemantics, and $\mathfrak {M}^+_p$ or $\mathfrak {M}^+_{gb}$ for presentist and growing block actualist presemantics respectively. The history-dependent TRL view thus consists of a model $\mathfrak {M}^+$ as per Definition 10 combined with the Priorian-Ockhamist semantics as per Definition 3 (suitably adapted to the new model).

Let me justify again in more detail why this is an adequate formal framework for LFOT. On the one hand, the Priorian-Ockhamist semantics generates the desired validities, and guarantees that we are able to give a sensible account of the future tense operator even at non-actual moments. This approach thus meets the second desideratum, formal adequacy. It is for this reason that it is preferable to the history-independent TRL of Sect. 5.

On the other hand, the $\hbox {TRL}_2$ model $\mathfrak {M}^+=\langle \Im , V, trl_h, C^t, I\rangle$ contains two elements that are individually necessary and jointly sufficient to secure the second desideratum, descriptive adequacy. Firstly, the BT-structure $\Im$ allows for a representation of the type of contingency required to represent free action. Secondly, ${trl}_h$ ensures that there is a *comprehensive* set of contingent propositional truths about the *actual* future. Here is how. The Priorian-Ockhamist semantics guarantees, as we have seen, a comprehensive set of contingent propositional truths about *this or that* future. What ${trl}_h$ does, then, is to specify a proper subset thereof that forms the set of comprehensive contingent propositional truths about the *actual* future, namely that proper subset whose members have ${trl}_h$ feature as the history-parameter of their point of evaluation. In other words, ${trl}_h$ breaks the stalemate over which moment/history pairs are the ones that describe what will *actually* happen. This second reason is what makes my hybrid view preferable to the postsemantic TRL of Sect. 6. One might at this point be tempted to introduce a new operator $F_@$ (“It will *actually* be the case that...”). However, this would just lead to new formal problems. So, seeing how my point can be made sufficiently well without positing a novel operator, I have refrained from doing so.

An example will help to illustrate the appropriateness of my framework more clearly. Suppose, with the proponent of LFOT, that you are free with respect to whether you will finish this paper. This means that there must be at least two possibilities open to you, namely the one in which you do, and an alternative one in which you don’t. Now, this multiplicity of possibilities is handled by a $\hbox {TRL}_2$ model $\mathfrak {M}^+$ in virtue of its allowing for branching. Let us therefore imagine a $\hbox {TRL}_2$ model $\mathfrak {M}^+$ consisting of two histories $h_1=\langle m_{0}, m_1, m_2\rangle$ and $h_2=\langle m_{0}, m_1, m_3\rangle$, designate $m_0$ as the present moment, use *p* as shorthand for “you finish this paper”, and stipulate that *p* is true at $m_2$ but false at $m_3$. Given Priorian-Ockhamist semantics, we thus wind up with two equally unprivileged truths, namely $\mathfrak {M}^+_{m_0,h_1}\vDash Fp$ and $\mathfrak {M}^+_{m_0,h_2}\vDash F\lnot p$. But the proponent of LFOT also believes that there is a privileged contingent propositional truth about what will *actually* happen. And this is handled by $\hbox {TRL}_2$ model $\mathfrak {M}^+$ in virtue of its containing an element ${trl}_h$ that tells us which of the previous two truths is the one that *actually* obtains. Let us stipulate that ${trl}_h=h_1$. In that case, the history-dependent truth about what will actually happen is $\mathfrak {M}^+_{m_0,h_1}\vDash Fp$, because only this truth features ${trl}_h$ as its history-parameter. Unlike in the case of the postsemantic TRL, the above holds for any *future-tensed sentence* *irrespective* of whether it is uttered or not – my framework thus doesn’t *under-count* the future truths. And unlike the history-independent TRL, the above can be maintained without any formal inadequacies. LFOT’s full picture is then finished off by adding the stipulation that God does not foreknow that $\mathfrak {M}^+_{m_0,trl_h}\vDash Fp$.

We have thus seen that my framework accurately represents temporal reality as LFOT suggests it to be. So it is time to now turn towards an objection. Historically, the literature has been *highly critical* of the idea of combining a history-dependent semantics with a model featuring a trl. Before surveying the reason, let me first say a bit more about what a mathematical model is. A useful starting point is the following definition (Bender, 2000, p.2):

**Mathematical models** A mathematical model is an abstract, simplified, mathematical construct related to a part of reality and *created for a particular purpose* (emphasis mine).

Naturally, a suitable model will therefore ideally consist only of elements that are individually necessary and jointly sufficient to fulfil its purpose.

TRL models were initially created as *semantic* models. Their purpose was to tell us how to assign history-independent truth-values to sentences (particularly future-tensed ones) (Wawer, 2014, p.377). Thus conceptualised, combining them with a history-dependent semantics would obviously be inappropriate. As Wawer (ibid.) correctly points out, not only would this betray the rationale of introducing a trl in the first place (which was to *eliminate* the history-parameter from the point of evaluation), but it would also rob the trl of any essential role in evaluating the future tense operator – to see this point clearly, it suffices to notice that the Priorian-Ockhamist semantics defines the truth conditions for future-tensed sentences *without* reference to the trl.

In order to be conceptually appropriate, the history-independent TRL must therefore be justified by reference to a different purpose. In the current context, the stated purpose is that of modelling LFOT. And, as we have seen, this purpose makes certain demands on formal and descriptive adequacy, demands that I have argued are met only by the history-dependent TRL. Specifically, I suggested that only the combination of Priorian-Ockhamist semantics and a trl is able to produce a plausible picture of the kinds of truths about the future the proponent of LFOT posits. So while it would indeed (as per Wawer) be inappropriate to use the history-independent TRL for purely *semantic* purposes, its use is perfectly appropriate for modelling temporal reality as posited by LFOT. Having thus presented a suitable formal model for LFOT and justified its legitimacy, it is now time to sharpen the second and third prongs of my philosophical defence of LFOT, i.e. to defend LFOT against its two most prominent objections.

## Todd’s grounding objection

### The objection

To set the stage, Todd (2014) makes the general point that for any response to a valid argument to count as a genuine *solution to* that argument, it has to do two things. Firstly, identify the faulty premise(s); and secondly, give an explanation of *why* a given premise is false. He then introduces a constraint on what shape such an explanation may take, namely that it cannot merely be that the relevant premise is logically incompatible given the negation of the argument’s conclusion (holding fixed the other premises). Another way of putting this is that a *Moorean-shift-style* response will always fall short of counting as a genuine solution.

Next, he correctly observes that the proponent of LFOT has only *identified* which part of the DFA she rejects (namely, the foreknowledge assumption), but that she has not explained *why* this assumption is false. Todd grants that there are ways of understanding LFOT on which it is not even *intended* to be a solution: one could, for example, interpret the proponent of LFOT as simply expressing scepticism about the foreknowledge assumption (*playing defence*, so to speak) without ever even trying to offer an explanation of its defectiveness. But the point remains that many have at least *taken it* to be a solution. The proponent of LFOT thus understood therefore owes us an explanation of why it is logically impossible for God to foreknow the relevant truths that goes beyond merely stating that foreknowledge is logically impossible *given* free will (and holding fixed the remaining premises). And this bring us to the crux of Todd’s objection, which is that the most plausible such explanation renders future contingent truths problematically *ungrounded*.

The explanation Todd proposes on behalf of the proponent of LFOT is that God *necessarily *only believes on *sufficient evidence*. Here, the *kind of evidence* that could both always have been available to God and sufficient to justify an infallible belief that *Fp*, Todd (ibid., p.532f.) surmises, would have to consist of the trends that make it *causally determined* that *p* will be the case. If the occurrence of *p* were thus determined, God could after all simply *deduce* the truth of *Fp* from His perfect knowledge of reality’s original state and causal tendencies. But the contingent future is *by definition* not causally determined. Consequently, since foreknowledge by necessity requires evidence, it is logically impossible for God to know about the contingent future because no such evidence is available. Et voilà – a suitable explanation of the foreknowledge assumption’s defectiveness.

But this solution, Todd maintains, precludes a suitable grounding account for future contingent truths. He worries that if there are truths that not even God is able to find grounds to believe, then plausibly there just *aren’t any grounds*. If true, this would conflict with the *grounding requirement* that truths be anchored in reality. Let $\phi$ be a nominal variable and *p* be a sentence letter. The grounding requirement can then be put as follows (Correia and Rosenkranz, 2022, p.121):

**Grounding requirement** For any truth $\phi$ of kind *K*, there is a *p* such that its being the case that *p* grounds its being the case that $\phi$ is true.

Denying (something like) the grounding requirement is problematic, Todd (ibid., p.533) argues, because doing so means one must reject the thesis that *truth supervenes on being*, i.e. the thesis that there cannot be a difference between two worlds with respect to what is *true* without a difference in what *exists*. And this just seems like an eminently plausible principle. Todd (2014, p.536) gives the following reason for taking LFOT to be in conflict with this thesis:

The way reality appears to God is the way reality is. But reality would appear to be the same to God under conditions that [$\phi$] and under conditions that [$\lnot \phi$] [or else He would have evidence for forming a belief either way]. So reality would be the same under conditions that [$\phi$], and under conditions that [$\lnot \phi$]. So there can be a difference in truth without a difference in reality. So truth does not supervene on being.

Consequently, Todd concludes that *if* LFOT is to be a solution to the DFA, and *if* the solution is to proceed along the lines he has sketched, *then* the proponent of LFOT must reject the thesis that truth supervenes on being – and this is bad. Meeting Todd’s challenge thus requires firstly the provision of a grounding account for future contingent truths, and secondly the imposition of suitable restrictions on divine cognition that explain why these truths nonetheless remain inaccessible.

### Two responses

I will present what I take to be the two most plausible such approaches. Before doing so, however, it must be made explicit that certain models of divine cognition are already incompatible with LFOT *irrespective* of any concerns about grounding. Both the *conceptualist* view of divine knowledge (see Craig, 1987, p.121ff.) according to which it is simply a conceptual truth that God essentially and innately knows all truths, and Fischer’s (2016, pp.31-45) *boot-strapping* account according to which God can *reason* Himself from fallible to infallible beliefs via self-knowledge of His infallibility, are non-starters here as they entail the existence of foreknowledge of the contingent future. I submit that this is not too damaging: after all, it is unclear to what extent the conceptualist view is an explanation *at all*, and there is reasonable doubt (see ibid., p.43f.) whether the boot-strapping view can explain why God would form any belief on the contingent future at all. My concern is therefore whether either of the following grounding accounts *adds* any additional constraints in this regard.

Arguably the most natural response to the grounding challenge is to adopt eternalism and ground future contingent truths in the eternal existence of the events they are about. This account accepts the grounding requirement, and tells us to fill in “*X* exist(s)” for *p* (where *X* denotes the relevant part(s) of future ontology that make $\phi$ the case). A suitable formal framework for this approach is given by a history-dependent $\hbox {TRL}_2$ model $\mathfrak {M}^+_e$. Todd (2014, fn.13) does anticipate this reply in a brief footnote; however, he declares that he is “supposing the proponents of LFOT will not want to be eternalists”, and that if a commitment to eternalism turned out to be a prerequisite for LFOT, many would find this “unwelcome". He then (justifiably) remarks that a full evaluation of this approach exceeds the scope of his paper.

*Most* of this evaluation I already carried out in Sect. 7 by showing that $\mathfrak {M}^+_e$ meets both desiderata for being an appropriate framework for LFOT. One question that remains, however, is whether positing an eternalist ontology to ground the relevant truths requires any further restrictions on divine cognition beyond the ones the proponent of LFOT is already committed to. And here, the answer may initially seem to be “yes”. If one takes this approach one will have to deny that God can just *see* (in some quasi-perceptual sense) the relevant future ontology that now grounds future contingent truths. A metaphor sometimes employed here is that of a “time-telescope”.

But a known problem with this mode of divine cognition is that it will likely involve *retrocausation*. This is because time-telescopes seem to make the perceived entities (in this case, *future* entities) the causes of the relevant beliefs. Consequently, the extent to which denying this mode of divine cognition is problematic is inversely proportional to one’s proclivity for accepting the possibility of retrocausation. But the proponent of LFOT, *qua* incompatibilist, plausibly denies retrocausation *already*. This is because accepting the possibility of retrocausation opens the incompatibilist up to classic objections against the DFA. Therefore, it seems like the time-telescope should join the ranks of the conceptualist and boot-strapping accounts of divine cognition as categorically unavailable to the proponent of LFOT. The answer to whether this grounding account requires any additional restrictions on divine cognition, upon reflection, thus turns out to be a “no” after all.

But maybe Todd is correct, and the proponent of LFOT will want to endorse non-eternalism. In this case, she should adopt either the history-dependent $\hbox {TRL}_2$ model $\mathfrak {M}^+_p$ or instead its cousin $\mathfrak {M}^+_{gb}$, and champion Correia and Rosenkranz’s (2022) *revolutionary strategy*. The key insight behind their approach is, as Rosenkranz (2012) puts it in earlier work, “that certain kinds of truths are true only courtesy of other kinds of truths and of what makes the latter true”. This approach harnesses the truth-value link between the present truth of a future contingent and the future truth of a corresponding sentence about what will then be the present such as to ground the present truth of the former in the future grounds of the latter (cf. Correia and Rosenkranz, 2022, p.123). Put simply, the idea is that for $F\phi$ to now be a grounded truth, it suffices that at some later time there *will* exist something that grounds $\phi$ – crucially, this thing need not *presently* fall within the scope of the most unrestricted existential quantifier. The revolutionary strategy thus tells us to fill in “*X* will exist” for *p* (where *X* denotes the relevant part(s) of future ontology that make $\phi$ the case). This strategy does not require placing any additional limitation on divine cognition. This is because the grounds it postulates for future contingents do not yet exist.

The proponent of LFOT thus has a choice. She can either be an eternalist, hope Todd was wrong in predicting she wouldn’t want to to be, and adopt an uncontroversial grounding account. Or she can be an non-eternalist, in which case she must commit herself to what I suspect is a slightly more controversial grounding account. Either way, Todd’s challenge can be met *without* posing any restrictions on divine cognition that the proponent of LFOT has not already bought into.

## Arbour’s fatalistic objection

Echoing Purtill (1988), Arbour (2013) argues that proponents of LFOT are committed to a *logical* problem of fatalism that exists independently of divine foreknowledge. This problem is said to arise from the conjunction of two theses the proponent of LFOT is committed to, namely the necessity of the past, and the *omnitemporality of truth* (i.e. the thesis that if *p* is true at any time, then *Fp* is true at all earlier times and *Pp* is true at all later times). While something like this worry arises for all advocates of true future contingents, it seems *particularly* worrying for the proponents of LFOT who *qua* incompatibilists are committed to some version of the necessity of the past in a way others might not be.

Arbour’s argument here goes something like this. Suppose the following proposition is an omnitemporal truth: “you read this paper today”. In that case, it was already true yesterday that you would read this paper today. Consequently, in order to be able to do otherwise than read this paper now, you must have the ability to act in a manner such that, were you to act that way, a proposition that was true yesterday would in fact have been false. But the past is necessary. So you do not have such an ability. Therefore, you cannot do otherwise than read this paper today, and hence do so unfreely.

This seems like a good argument. So, given that it is impossible for the proponent of LFOT to deny the omnitemporality of truth (after all, she is essentially committed to there now being truths about the future), she must deny an *unrestricted* version of the necessity of the past. But care is required here. After all, some relevant conception of the necessity of the past is required to motivate her incompatibilism about foreknowledge and freedom. Arbour’s objection is thus most fruitfully understood as a challenge to devise a *restricted* principle of the necessity of the past that *validates* the DFA but *invalidates* the parallel argument for logical fatalism.

On the formal side, the framework I proposed in Sect. 7 heeds the requirement that there be no unrestricted principle of the necessity of the past. Such a principle can formally be represented by the schema $P\phi \rightarrow \Box P\phi$. Fortunately, it is invalid in Priorian-Ockhamist semantics. To see why, consider again the $\hbox {TRL}_2$ model consisting of two histories $h_1=\langle m_{0}, m_1, m_2\rangle$ and $h_2=\langle m_{0}, m_1, m_3\rangle$, and stipulate that *p* is true at $m_2$ but false at $m_3$. In this case, at the pair $m_1/h_1$, $PFp$ is true but $P\Box Fp$ is false.

The first step towards meeting Arbour’s challenge, then, is one I already took in Sect. 2 by predicating accidental necessity of *events* rather than *propositions*. Equipped with this understanding of the necessity of the past, the argument from logical fatalism as presented above fails. This is because it required propositions to be accidentally necessary.

However, the argument can easily be rephrased in terms of events instead. Suppose that instead of the proposition *that you read this paper today* we imagine the event of you having yesterday uttered the following promise: “I will read this paper tomorrow”. Now, as you are currently reading this paper, it follows from the omnitemporality of truth that you spoke truly yesterday. In order to be able to not read this paper, you therefore require an ability such that, were you to exercise it, a true utterance in the past would instead have been false. This, Arbour (2013) rightly points out, plausibly counts as a power over the past. He then contends that the proponent of LFOT denies we have any power over the past. Ergo, she is stuck with fatalism after all, or so the revised argument goes.

At this point, it seems obvious to me that the proponent of LFOT *must* allow for the sort of power Arbour alleges she denies, namely, the power to act in a way such that, were one to act that way, some true past utterance or belief (or any event involving a commitment of sorts to a proposition) would instead have been false. Now, one might be tempted to think that this undermines LFOT’s commitment to theological incompatibilism: after all, if I have such a power, then why can’t I make it the case that God held a false belief in the past? Indeed, I suspect this concern is what motivated Arbour to insist the proponent of LFOT cannot accept the relevant power.

But the answer here is simple. The ability to act in a way such that, were you to act that way, God would have held a false belief in the past, is an ability that is ruled out by considerations wholly *orthogonal* to the necessity of the past. To see this, one only need to consider the analogous future-directed version of this ability, which is just as impossible. The issue here is thus not one of temporal necessity, but *logical impossibility*. To the extent that the proponent of the DFA defended this impossibility with respect to temporal concerns, she should admit that she has made a mistake, and accept this new justification instead. Crucially, one can accept the relevant power over the past without this threatening entailing a power to make God have held a false belief in the past. And the same should be said about the ability to act in a way such that, were you to act that way, God would not have existed. Again, this ability seems to be ruled out by more than just temporal concerns.

The only point of the DFA at which a conception of the necessity of the past is required is the contention that we lack the ability to act in a way such that, were we to act that way, God would have held a *different* belief in the past. And all that is required here is the necessity of the past as outlined in Sect. 2, a principle that is perfectly compatible with the kind of power required to avoid logical fatalism. All is well then for the proponent of LFOT: she can avoid the threat of logical fatalism by allowing for a certain limited power over the past without thereby calling into question her theological incompatibilism.

## Conclusion

This paper mounted a three-pronged philosophical defence of LFOT. On the one hand, I showed that the view could be formalised in a both formally and descriptively adequate way. Doing so, I argued, required combining a $\hbox {TRL}_2$ model with standard Priorian-Ockhamist semantics, a move that has historically been shunned. I then provided good reason for deeming this combination conceptually appropriate. On the other hand, I harnessed the insights gained by this formalisation in order to respond to the two most prominent objections to LFOT, namely Todd’s (2014) grounding objection and Arbour’s (2013) fatalistic objection. With respect to the former, I showed that LFOT could be a solution to the DFA without running into grounding problems. As regards the latter, I demonstrated that the proponent of LFOT can posit some limited power over the past sufficient to avoid logical fatalism without it threatening her acceptance of the DFA. Consequently, we should consider the possibility that it was always contingently true that LFOT would be rehabilitated *despite* God never foreknowing this.

## Footnotes

1 I apply this label based on the admittedly somewhat arbitrary criterion that these are the only two objections to be directly referenced in the relevant entry of the Stanford Encyclopedia of Philosophy (see Hunt and Zagzebski, 2022, §2.2).

2 I am going to assume that God holds beliefs *at times* and draw no distinction between divine *belief* and divine *knowledge*.

3 It does not cover, however, views which treat God as being in some sense *outside of time.*

4 This assumption becomes obvious when Wawer (2014, p.383) states that on “the branching concretist picture, every context initializes a single possible moment, namely *itself*” (emphasis mine), and when he states that, on some views of temporal ontology, there is an “isomorphism” between *C* and *M* (or parts thereof).
